# Transcatheter arterial embolization with *N*-butyl cyanoacrylate for arterial esophageal bleeding in esophageal cancer patients

**DOI:** 10.1186/s12957-016-0803-y

**Published:** 2016-02-24

**Authors:** Makoto Aoki, Hiroyuki Tokue, Yoshinori Koyama, Yoshito Tsushima, Kiyohiro Oshima

**Affiliations:** Department of Emergency Medicine, Gunma University Graduate School of Medicine, Maebashi, Gunma Japan; Department of Diagnostic and Interventional Radiology, Gunma University Graduate School of Medicine, Maebashi, Gunma Japan

**Keywords:** Esophageal cancer, Transcatheter arterial embolization, *N*-butyl cyanoacrylate

## Abstract

**Background:**

The aim of this study is to evaluate the clinical efficacy and safety of transcatheter arterial embolization (TAE) with *N*-butyl cyanoacrylate (NBCA) for the treatment of arterial esophageal bleeding in esophageal cancer patients.

**Methods:**

Between November 2008 and December 2014, five esophageal cancer patients underwent TAE with NBCA for the treatment of arterial esophageal bleeding. We retrospectively evaluated the technical and clinical success, recurrent bleeding, and procedure-related complications.

**Results:**

All of the patients had bleeding from the esophageal artery and were in shock at the beginning of TAE. Four patients had a coagulopathy at the time of TAE; however, the TAE could successfully arrest bleeding in all five patients. After TAE, they immediately recovered from the shock state. Two patients were discharged without event, one patient is currently hospitalized for another complication, and the other two patients died due to multiorgan failure. In addition, no procedure-related complications such as esophageal infarction and recurrence of arterial esophageal bleeding were observed during this study.

**Conclusions:**

TAE with NBCA can arrest bleeding in esophageal cancer patients with active arterial esophageal bleeding, even in those with a pre-existing coagulopathy.

## Background

Esophageal cancer patients may suffer lethal esophageal artery bleeding [1]. Conventionally, the traditional first line of therapy for intraesophageal bleeding from the esophageal artery is an endoscopic procedure [2] and that for intrapleural bleeding is surgery [3]. However, with the advancement of technology, transcatheter arterial embolization (TAE) of the esophageal artery can be an effective alternative treatment [4–7]. It was reported that TAE was useful for the treatment of endoscopically unmanageable non-variceal upper gastrointestinal bleeding, and there was some possibility of rebleeding due to coagulopathy [8].

*N*-butyl cyanoacrylate (NBCA) has been used for many years as a liquid embolic material and was demonstrated to be useful for various diseases and conditions [9–16]. An advantage of TAE with NBCA is its high success rate of occlusion even in patients with a coagulopathy; however, there are disadvantages that NBCA could cause ischemic injury and is difficult to handle precisely [17].

We report the clinical efficacy and safety of TAE with NBCA for treatment of arterial esophageal bleeding in esophageal cancer patients.

## Methods

### Patients

The protocol of this retrospective study was approved by the research ethics board of the Gunma University Hospital without the need for informed consent.

Between November 2008 and December 2014, five patients underwent TAE for the treatment of arterial esophageal bleeding at our institution. All of the patients were male, ranging in age from 66 to 77 years (mean age, 71 years). All patients had esophageal cancer. Four patients had undergone surgery for esophageal cancer, and the fifth patient had undergone photodynamic therapy (PDT). Patients who had undergone surgery developed arterial esophageal bleeding 1 day, 10 days, 3 months, and 8 years after the surgery, respectively. The patient who had undergone PDT developed arterial esophageal bleeding 2 days after the PDT.

### Procedure

We used NBCA (Histoacryl; B. Braun, Melsungen, Germany) as the embolic material in all five patients. NBCA was chosen because the majority of patients had a coagulopathy and were in an unstable condition. Before angiography, if possible, we performed enhanced computed tomography (CT) to confirm the extravasation and the location of the esophageal artery; if it was not possible to perform enhanced CT, we checked previously obtained enhanced CT scans. We performed diagnostic angiography to localize the bleeding site after common femoral artery puncture. If we failed to find the bleeding site on aortography, we performed selective angiography of the esophageal artery directly from the aorta with a 5-Fr Michelson catheter (Medikit Co. Ltd., Tokyo, Japan), intercostal artery, bronchial artery, or left inferior phrenic artery. After confirming the active bleeding site, we advanced a microcatheter (Estream 2.0; Toray Medical Co. Ltd., Tokyo, Japan) as close as possible to the bleeding site in the esophageal artery. Next, we mixed NBCA with iodized oil (Lipiodol; Andre Guerbet, Aulnay-sous-Bois, France) at a ratio ranging from 1:1.5 to 1:3. Prior to injection of the NBCA mixture, we flushed the microcatheter with 5 % dextrose solution to prevent premature polymerization of the mixture within the microcatheter. We injected the NBCA mixture using a 2.5 mL syringe under fluoroscopic monitoring. Next, we performed post-embolization angiography to evaluate the effectiveness of the treatment.

We retrospectively evaluated the technical and clinical success of TAE, recurrent bleeding, procedure-related complications, and clinical outcomes of each patient. We defined technical success as successful superselection of the bleeding vessel and delivery of the NBCA mixture, as well as an angiographic result after embolization that showed no evidence of active bleeding (i.e., the presence of a pseudoaneurysm or extravasation of the contrast agent). We defined clinical success as clinical improvement without evidence of bleeding (i.e., clearing of the nasogastric aspirate and stabilization of the hemoglobin level). Patients who met one of the following criteria were considered to have a coagulopathy: a prothrombin ratio of greater than 1.5, a partial thromboplastin time of greater than 45 s or a platelet count of less than 80,000/μL. Complications were evaluated according to the Common Terminology Criteria for Adverse Events v4.0 (CTCAE).

## Results

**Fig. 1:**
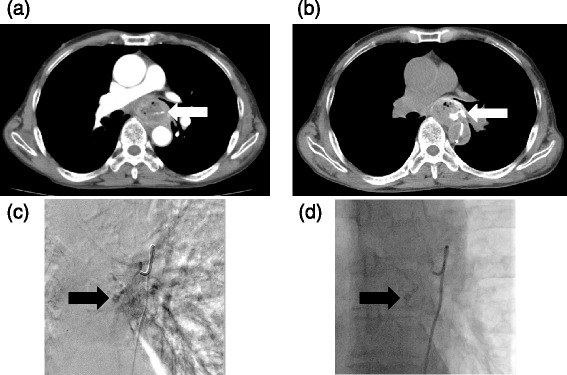
Arterial esophageal bleeding in case 2. **a** Enhanced CT shows that contrast material was in the esophagus in the arterial phase, indicating extravasation of contrast material (*arrow*). **b** Enhanced CT with angiography of the esophageal artery shows contrast material extravasation in the esophagus (*arrow*). **c** An image of digital subtraction angiography of the esophageal artery demonstrates punctate contrast material collections in the esophagus (*arrow*). **d** After transcatheter arterial embolization with a mixture of *N*-butyl cyanoacrylate (NBCA) and Lipiodol (*arrow*), active bleeding improved

The clinical and angiographic data of the five patients with arterial esophageal bleeding are summarized in Table 1. The example case is shown in Fig. 1. We diagnosed four of the five patients with coagulopathy from hemorrhagic shock at the time of embolization. Before TAE, enhanced CT was performed in all patients. In the four patients, the esophageal artery was identified as a branch of the aorta by referring to a past CT scan. All the patients had a bleeding esophageal artery that originated directly from the aorta. Two of the five patients had an esophageal artery that originated in a common trunk with the right bronchial artery. The angiographic findings in the four patients indicated extravasation of the contrast agent, and two of the five patients had pseudoaneurysm of the esophageal artery. After embolization, each patient showed clearing of the nasogastric aspirate and stabilization of the hemoglobin level without additional transfusion, and in turn, achieved technical and clinical success. Further, there was no evidence of symptomatic esophageal infarction or other major complications directly related to NBCA embolization in any of the patients. One patient suffered cardiopulmonary arrest before TAE The patient could be resuscitated and TAE was completed; however, he died due to multiorgan failure 6 days later. Another patient died due to other underlying conditions. This patient developed sepsis and multiorgan failure 2 months after TAE. The other three patients continue to undergo regular follow-up and they have had no complications from TAE to date.

**Table 1:** Clinical and angiographic data of five patients with arterial esophageal bleeding

No.	Age (year)	Staging	Origin of esophageal artery	Treatment progress of esophageal cancer	Coagulopathy	Bleeding site	Angiographic finding	The ratio of NBCA/Lipiodol	Result of admission
1	70	pT1bN3M0	Aorta (right bronchial artery)	Post-operation radiation 3 months	Present	Right pleural, mediastinum	Pseudoaneurysm	1:3	Died due to multiorgan failure 2 months later
2	66	cT3N1M1	Aorta (right bronchial artery)	Pre-operation radiation day 2	Present	Esophagus	Extravasation	1:2	Discharged without event
3	75	pT1bN0M0	Aorta	Post-operation 8 years	Present	Esophagus, right pleural, peritoneal, mediastinum	Extravasation and pseudoaneurysm	1:1.5	Died due to multiorgan failure 6 days later
4	77	pT3N3M0	Aorta	Post-operation day 1	Present	Right pleural, peritoneal, mediastinum	Extravasation	1:3	Discharged without event
5	68	pT3N1M0	Aorta	Post-operation day 10	Absent	Mediastinum	Extravasation	1:3	Discharged without event

All of the patients were male

## Discussion

NBCA has been used for many years as a liquid embolic material and was demonstrated to be useful for occlusion of cerebral arteriovenous malformation and fistula [9], hemorrhage of visceral arteries [10–13], percutaneous transhepatic portal embolization prior to partial hepatectomy [14], varicocele embolization [15], and type 2 endoleak embolization after stent-graft repair [16].

NBCA is a monomeric liquid adhesive that polymerizes in the presence of ionic solution, such as blood. It polymerizes rapidly and forms a glue cast that strongly adheres to the tissue. The polymerization time of NBCA is controlled by addition of Lipiodol [18, 19], and it was reported that some degree of mural inflammation due to the embolization with NBCA to act in synergy with the embolizing substance to render permanent occlusion [20].

An advantage of TAE with NBCA is its high success rate of occlusion even in patients with a coagulopathy. Several reports have described the relationship between coagulopathy and the outcome of transcatheter arterial embolization with particles and coils for acute intestinal bleeding [10, 21–23]. In the article by Jae et al. [24], the clinical success rate of TAE in the group of patients with coagulopathy was 83 % (15/18). They concluded that TAE with NBCA is a highly effective and safe treatment modality for nonvariceal upper gastrointestinal bleeding, especially when it is not possible to advance the microcatheter to the bleeding site and when the patient has a coagulopathy [24].

Among our cases, hemorrhage from the esophageal artery was confirmed in the intraesophageal region in two cases and in the intrapleural region in four cases. One patient was scheduled to undergo esophageal cancer surgery and was receiving PDT. The most common significant adverse event after PDT is esophageal stricture formation [25], hemorrhage induced by PDT has not been reported.

The other patients were postoperative when arterial esophageal bleeding occurred. Out of four patients, two patients had esophageal bleeding in early postoperative period and were confirmed the increase of bloody drainage in chest tube. The obvious reason for esophageal bleeding was not clear; however, out of five patients, two patients had radiation therapy and we thought radiation therapy could be a risk for esophageal bleeding in preoperative and postoperative period. Treatment of esophageal bleeding with TAE with NBCA in patients with postoperative esophageal cancer has not been reported. The typical complications of surgery such as hemorrhage cannot be completely eliminated, and severe hemorrhage is a rare and potentially lethal complication after esophagectomy [1, 26].

In general, the traditional first-line treatment for hemorrhage in the intraesophageal region is an endoscopic procedure [2]. In our cases, two patients had hematemesis and one patient underwent the endoscopic procedure; however, the esophageal bleeding could not be controlled. Postoperative intrathoracic hemorrhage after esophagectomy is usually treated by reoperation. Indications for emergency thoracotomy for hemothorax include the following: bleeding through the chest drain at >100 ml/h for ≥5 h, or inability to maintain a normal blood pressure without blood transfusion [3]. Generally, reoperation is indicated; however, it is often difficult to reoperate due to the patient’s condition such as shock, disseminated intravascular coagulation, and so on. Among our cases, two patients had bleeding in early postoperative period and the management for the bleeding were discussed between surgeons and interventional radiologists, and TAE was selected as less invasive method. A criterion for TAE in patients with hemothorax is that extravasation is confirmed on contrast-enhanced computed tomography [27]. Among our cases, extravasation was confirmed by contrast-enhanced computed tomography before TAE in four cases and the differentiation between venous and arterial bleeding was done with it. Before the report by Park et al. [17], there were a few case reports about embolization treatment for the esophageal artery [4–7]. Similar to the result by Park et al., we succeeded in treating all the five patients, although four patients had an underlying coagulopathy at the time of the TAE.

Prior to performing emergent TAE, it is important to find information about the arterial blood supply of the esophagus. Branches of the subclavian, thyroidea ima, common carotid, or superior thyroid arteries may also supply the cervical esophagus. Branches of the right third or fourth intercostal arteries may also supply the midthoracic esophagus. Lastly, branches from the celiac, splenic, short gastric, or left hepatic arteries may supply the distal esophagus [5, 28]. About the patients with esophageal cancer, by enhanced computed tomographic scans, how much the esophageal artery was preoperatively identified has not been reported. Among our cases, the esophageal artery was identified with past computed tomographic scans in two cases. Before TAE, interventional radiologists can predict from where the bleeding esophageal artery originates by looking at past computed tomographic scans.

Our study has several limitations. First, the study population was small because arterial esophageal bleeding in esophageal cancer patients is very rare and this study was retrospective. Second, the observation period was short and we cannot predict the long-term prognosis. Third, this study was not organized as the comparative study between TAE with NBCA and TAE with other embolic materials.

With the advancements in microcatheters and embolic agents, TAE is becoming more important and effective in the treatment of hemorrhagic diseases. At present, it is not well known that esophageal bleeding in esophageal cancer patients can be managed by TAE.

## Conclusions

We reported the clinical efficacy and safety of TAE with NBCA for the treatment of arterial esophageal bleeding in esophageal cancer patients. We suggest that TAE with NBCA can be an effective option for arterial esophageal bleeding in esophageal cancer patients whether the hemorrhage is in the intraesophageal region or the intrapleural region. Interventional radiologists, surgeons, and emergency physicians should be aware of the usefulness of TAE with NBCA to arrest bleeding from the esophageal artery.
